# Predictors of voluntary uptake of modern contraceptive methods in rural Sindh, Pakistan

**DOI:** 10.1371/journal.pgph.0002419

**Published:** 2024-04-04

**Authors:** Zahid Memon, Abeer Mian, Wardah Ahmed, Muhammad Jawwad, Shah Muhammad, Abdul Qayyum Noorani, Zulfiqar Bhutta, Hora Soltani

**Affiliations:** 1 Aga Khan University, Karachi, Pakistan; 2 Aga Khan Foundation, Islamabad, Pakistan; 3 Sheffield Hallam University, Sheffield, England, United Kingdom; PLOS: Public Library of Science, UNITED STATES

## Abstract

The use of modern contraceptive methods (MCMs) has been stagnant for the last decade in Pakistan. The second most populous province, Sindh reports 25% of MCMs use. Various factors including demographics and health services utilization are associated with the uptake of family planning services. This research aimed to identify and assess specific predictors of MCMs among women aged 15–49 in two districts of Sindh-Matiari and Badin. A cross-sectional household survey was conducted from October 2020- December 2020. In total, 1684 Married Women of Reproductive Age (MWRA) 15–49 years were interviewed. For the selection of eligible respondents, a two-stage stratified cluster sampling strategy was used. Univariate and multivariable logistic regression was used to determine the predictors for the use of MCM. Use of modern methods of contraceptive was 26.1% (n = 441). Statistically significant socio demographic predictors of MCM included: number of children 4 or more (AOR: 5.23; 95%CI: 2.78–9.84), mother having primary education (AOR: 1.73; 95% CI: 1.26–2.36), and husband having middle education (AOR: 1.69; 95% CI: 1.03–2.76). Maternal health services indicators included: postnatal care of mother (AOR: 1.46; 95% CI: 1.09–2.05); women who were visited by Lady Health Workers in their postnatal period and were counselled on family planning (AOR: 1.83; 95% CI: 1.38–2.42). Since the primary purpose of using modern contraceptive methods is for limiting pregnancies, there is a potential to promote awareness about the benefits of birth spacing as part of implementing a more integrated approach to family planning. The integration of family planning services within maternal and newborn child healthcare services effectively promote the voluntary adoption of modern contraceptive methods. The role of Lady Health Workers in family planning counseling and service provision and uptake is important in the context of Sindh and should be fostered further by opportunities for capacity building and their empowerment.

## Introduction

The uncontrolled population in developing regions over the last decade has brought family planning and the advocacy for modern contraception to the forefront of the global agenda. Modern contraceptive methods (MCM) as defined by Hubacher et.al. refers to technological products or medical procedures that affect natural reproduction. They can be classified as short-acting reversible contraception (SARC), long-acting reversible contraception (LARC), or permanent methods [1]. Access to modern contraception is now recognized as a universal human right that significantly improves maternal and child health status, reduces poverty, and enables women’s economic participation [2]. Despite the investment in family planning through the initiation of policy and programs and subsequent gains in contraceptive uptake, approximately 210 million women of reproductive age residing in low-middle income countries (LMICs) still report an unmet need for contraception. This is due to limited access to contraceptive services both geographically and financially, poor quality of services, lacking agency and decision-making on reproductive health and misconceptions regarding the use and side-effects of contraceptive methods [3, 4].

Within Asia, Pakistan, with its annual growth rate of 2.5, has already exceeded South Asia’s average growth rate of 1.2% and is forecasted to catapult to 403 million inhabitants by 2050 [5, 6].

Pakistan initiated commitment to improving maternal and child health in 1994 through the launch of the Lady Health Worker Program—the flagship Family Planning program. Since the program’s launch, the country has become a member of the global family planning community and a signatory to FP 2023 commitments envisioning the empowerment of women and young couples by achieving a balance between family size and resources. A recent development in this context is the introduction of the Costed Implementation Plan (CIP) by the Sindh government under the FP2020-30 initiative [7] which holds the potential to expedite advancements towards achieving the desired modern contraceptive prevalence rate (mCPR) targets. The CIP was developed to mark out a strategic multi-year roadmap to achieve family planning goals. The country also remains committed to the fulfilment of the SDG goals (particularly Goal 3 Health and well-being) with a firm stance on ensuring comprehensive access to family planning resources, services, and commodities. Diverse initiatives have been initiated within the country in collaboration with international and domestic non-governmental organizations, with sustained support from National, Provincial, and District governments.

Despite these efforts and commitments, utilization of family planning services and uptake of contraception have been low as reflected in a marked by a country-wide CPR of 34%, and 25% of unmet need for MCM [8–10]. Within Sindh, the second most populous largest province of Pakistan Sindh reports, contraceptive use is only at 28.9%, of which 25% is attributed to MCMs use [5]. A review of the local literature on family planning and contraception reveals limited method choice, specifically for modern methods; social stigma attached to birth control; family and societal disapproval; and lack of knowledge of the importance of birth spacing and maternal health as have been as persistent demand side barriers [11]. On the supply side, irregular supply of contraceptive methods, poor attitude and competency of healthcare providers and limited coverage and outreach of family planning services, have been reported as significant factors that negate uptake [11–13]. These low rates of uptake, of any type of contraceptive methods, are dangerously manifested in the country’s high fertility rate of 3.56 births per woman, combined with a maternal mortality rate of 186/100,000 livebirths and under-5 mortality rate of 63.3 deaths/ 1000 live births [14, 15]. Besides mortality, concomitantly high rates of early marriages of girls before 18 years of age, unintended pregnancies and resulting abortions, estimated at annual rate of 50 per 1000 women [16] coupled with socio-cultural and patriarchal barriers, present a concerning picture of the sexual and reproductive health and overall well-being of women and young girls in the country.

The reality and magnitude of these manifestations placed against the country’s continued sub-national and national efforts towards family planning and enhancing contraceptive uptake, require an assessment into why there is a disconnect and more importantly, what is not working. This is an important gap that provides the rationale for this study as it attempts to uncover and study various predictors in the voluntary uptake of modern contraception, specific to the context of Pakistan that can explain and strategically address this gap.

### Aim

The main aim of this paper is to assess predictors associated with the uptake of modern contraceptive methods in the domains of sociodemographic, reproductive, and maternal health care with corresponding knowledge, and use amongst married women of reproductive age (MWRA) between 15–49 years in two districts of rural Sindh.

## Methods and materials

**Fig 1:**
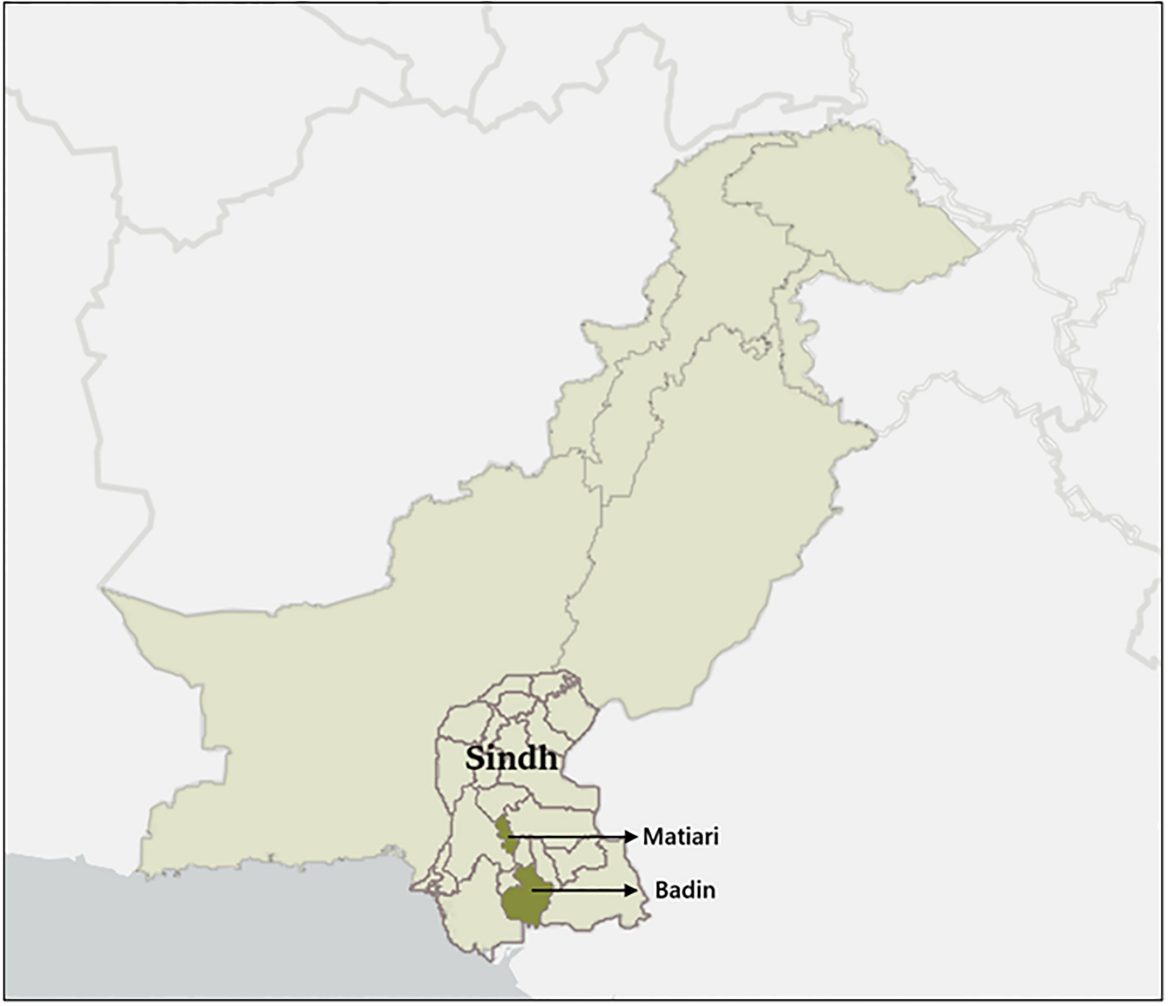
*Study Sites–Badin and Matiari districts of province Sindh, Pakistan. *Map developed on ArcGIS, available here for proper citation of base map: https://support.esri.com/en-us/knowledge-base/faq-what-is-the-correct-way-to-cite-an-arcgis-online-ba-000012040#:~:text=To%20reference%20the%20use%20of,Scale. Compatible with our CC-BY 4.0 license.

This was a cross-sectional study with face-to-face interviews of married women of reproductive age at household level, using a structured questionnaire, as the pregnancies in Pakistan primarily occur following marriage [17]. This survey was conducted in two districts of the Sindh province, Matiari and Badin (Fig 1).

The selection of districts was based on propensity matching score further details could be obtained from protocol paper [18]. PSM is a statistical method to create a propensity score for participants based on the variables of interests or covariates among the districts. PSM also reduces the confounding biases in observational studies thereby decreasing challenges in comparison of the respondents from two population [19]. In this research, the matching process involved a sampling frame that included 23 districts within the Sindh province, utilizing data from the Multiple Indicator Cluster Survey (MICS) conducted in 2018 [5]. Data was extracted on all relevant program indicators, including the study population’s mCPR, antenatal and postnatal care services, facility births, and socio-economic and demographic status. Detailed PSM scores found in the supporting information file (S1 Table). The final district selection was approved by the Director General (DG) Health Sindh and project lead consultative meetings.

### Sample size estimation

As per the Multiple Indicator Cluster Survey for Sindh 2018–2019 [5], the CPR reported for Sindh province was 28.9%. Based on the CPR, with 95% CI and 80% power, an assumed design effect of 1.5 and a 7% non-response rate, a sample size of 880 MWRA (15–49 years) per district was estimated.

The targeted population for this study consisted of MWRA between 15–49 years of age residing in the study districts within the catchment population of public sector health facilities. Currently pregnant, women who have had hysterectomy and menopausal women were included in study to record their reproductive history. The exclusion criteria for the sample included: (i) families that recently relocated to the project area within three months, with no intention of residing there for an extended period and (ii) women of reproductive age with mental disorders.

A two-stage stratified cluster sampling strategy was used to select eligible respondents. Clusters were created with at least 100–150 households and were established as primary sampling units (PSUs). In the first sampling stage, clusters were randomly selected from each district’s list of clusters (sampling frame). Subsequently, a line listing operation was conducted to determine eligible households assigned as secondary sampling units (SSUs). In the second stage, 20 eligible households with at least one MWRA were selected using systematic sampling to reach the calculated sample size (covering 44 clusters in each district).

### Survey questionnaire

The validated household questionnaire was adopted from the Pakistan Demographic and Health Survey (PDHS) questionnaire for women of reproductive age [8]. The questionnaire was then pre-tested in 5% of the total sample population, then translated into the local Sindhi language. The PDHS questionnaire was a reliable instrument at population level settings for assessing reproductive health of women and socio-demographic characteristics of the household in the area. For this the internal consistency measured through Cornbach’s alpha, and the value found to be 0.75 which showed tool was fulfilling the psychometric property of reliability. A customized digital application and software were developed for digital data collection.

### Survey training

Before data collection, a five-day training workshop on the data collection process was conducted for field teams (directly involved in field-based data collection). All sections of the questionnaires were discussed in detail, followed by a mock field-based data collection activity for data collectors supervised by the senior research staff. Training manuals were developed and distributed to the data collectors and questionnaires for pilot testing in the field.

### Data collection and data confidentiality

The study was conducted in each district by the assigned data collection team consisting of one team leader and four data collectors. Before the data collection process commenced, line listing was undertaken in selected clusters. Verbal informed consent was taken from married respondents and details of the risks and benefits of their participation were explained in the presence of an impartial witness and guardian if respondent was under 18 years of age. They were given an opportunity to ask any questions they had regarding the study, after which their consent was obtained (except for those refusing to participate). As the population-based household survey considered a minimal risk with maintaining anonymity using unique identifier, obtaining verbal consent may be sufficient [20]. Trained data collectors read aloud the simplified informed consent and assent in local language (Sindhi) to aid in understanding of all the components of consent with the consideration of patient comprehension [21]. The data collector records the verbal informed consent and verbal assent by recording the “agreement to participate”, in the presence of head of household, witness and guardian who was at least 18 years of age [21]. The process of consent was conducted under the supervision of the team lead. Data was collected between 1^st^ October 2020 and 31^st^ December 2020, on personal digital assistants (Model Samsung SM T289) through an Android application designed for field-based data collection. Data collection duration was 45–60 minutes. All data was uploaded electronically, analyzed, and displayed on the virtual project dashboard. To maintain confidentiality, coded data was stored in a password-protected and encrypted system, ensuring that only authorized individuals had access to it.

### Measurements

#### Outcome (dependent) variable

The primary outcome variable of interest was Modern Contraceptive Methods (MCMs). The use of modern methods of contraceptives was measured as a dichotomous dependent variable that takes the value of one if the woman (or partner) is currently using any modern method; or then zero in any other case. The short-term methods include pills, injectables, condoms, standard days methods (SDM), lactation amenorrhea method (LAM). The long-acting reversible contraceptives (LARC) include intrauterine devices (IUCD) and implants whereas female sterilization, male sterilization classified as irreversible/permanent contraceptive methods. The secondary outcome was ever use of modern or traditional contraceptive method and knowledge of any one of the contraceptive methods.

#### Predictor (independent) variables

Previous literature suggests various predictors of voluntary uptake of MCM [8, 9, 22]. Predictors included sociodemographic variables, including the household size that’s the number of household members living in the house for more than three months. Respondent’s age in years, number of living children, respondent’s education and her husband’s education categorize as no education, primary, middle, secondary and intermediate or above. Additionally, the wealth quantile was used as an indicator of the distribution of wealth in the population covered. A wealth index was constructed using principal components analysis and predicted using the first principal component. The index was calculated at the household level and based on the description in demographic health survey (DHS) survey procedures [23]. The score was categorized into five equal quintiles, namely poorest, poorer, middle, richer, and richest, with the first quintile representing the poorest 20% and the fifth quintile representing the richest 20% of the population.

Maternal health care variables included: women who sought antenatal care, four or more antenatal care visits and postnatal care visits (within 48 hours of home and facility-based delivery) at health facility level, skilled birth attendant, type of delivery as normal or cesarean sections and had received advice for family planning methods from Lady Health Workers (as part at their routine visit in during pregnancy and postnatal care visit).

### Data analysis

The collected data was then analyzed in STATA version 17 (Stata Corp, Texas). Descriptive statistics and inferential statistics used to summarize the data. Categorical variables were presented in frequency and percentages, while mean and standard deviation were used for continuous variables. Furthermore, univariate and multivariable logistic regression was used to determine the predictive factors associated with increase in the use of modern methods of contraceptives amongst married women of reproductive age. Variables significant at a p-value of less than 0.20 in the univariate analysis were included in multivariable logistic regression to measure association at 95% confidence interval [24]. This relies on the Wald test derived from logistic regression, with a p-value threshold set at 0.2. Conventional levels like 0.05 may fail in the identification of variables acknowledged as significant. The final model was constructed using backward elimination; variables were retained if the p-value was less than 0.05. During the iterative variable selection process, non-significant covariates that are not confounders are eliminated from the model [24]. The multicollinearity was checked using Varian Inflation Factor (VIF). The VIF values for all variables used after univariate model with p value <0.2 in the adjusted model was less than 5 (mean VIF 1.5). However, there was multicollinearity between facility birth and skilled birth attendant (mean VIF of 11.3). The variables used in the final adjusted model were less than 5, which indicates that multicollinearity is not a problem in the regression model (mean VIF 1.38).

### Ethical approval

Ethical approval was received from the Ethical Review Committee (ERC) of the Aga Khan University on July 16, 2020 (2020-3606-18261). The study protocol was approved by the National Bioethics Committee, Pakistan (4-87/NBC-514/22/857). Study questionnaire, informed consent and assent was approved by ERC- AKU.

## Results

From the total sample size of 1,760, 96% (n = 1,684) of MWRA were interviewed. There was a high response rate of 96%, as projected in the interview-based studies at the household level.

### Sociodemographic characteristics of MWRA

In terms of sociodemographic characteristics in Table 1, the mean age of the study participants was 32.3years, with a standard deviation (SD) of 7.1 years. Among respondents, 27.0% (n = 453) had formal education. The average number of children per woman was 4.0± 2.0, and the average number of members living in the household was 7.2 ± 2.7.

**Table 1:** Socio demographic characteristics of MWRA(n = 1,684).

Variable	Overall	
	**mean**	**SD**
**Household Size**	7.2	±2.7
**Respondent’s age**	32.3	± 7.1
**Husband’s Age**	36.4	± 8.5
**Number of Living Children**	4.0	±2.0
	**n**	**%**
**Respondent’s Education**		
No education	1231	73.0
Primary	224	13.3
Middle	58	3.4
Secondary	68	4.0
Intermediate or above	103	6.1
**Husband’s Education**		
No education	659	39.1
Primary	325	19.2
Middle	109	6.4
Secondary	213	12.6
Intermediate or above	357	21.1
**Wealth Quantile**		
Poorest	335	19.8
Poor	336	19.9
Middle	339	20.1
Rich	338	20.0
Richest	336	19.9

### Current use of contraceptives

Table 2 showed that from a total of 1,684 married women, 76.2% (n = 1255) reported that ever heard of at least one contraceptive method to delay pregnancy.

**Table 2:** Current use of contraceptives reported by MWRA (n = 1,684).

Characteristics	Overall	
	n	%
**Ever heard of at least one method to delay pregnancy**	1255	76.2
**Ever heard of at least one long term method to delay pregnancy**.	890	52.8
**Ever used any method to delay/avoid pregnancy**	625	38.5
**Current use of contraceptive**	463	27.4
**Any modern method**	441	26.1
Pills	83	5.0
Injectables	63	3.7
Condoms	87	5.1
Intra Uterine Devices(IUD)	12	0.7
Implant	41	2.4
Standard Days Method (SDM)	1	0.1
Lactational Amenorrhea Method (LAM)	6	0.3
Female sterilization	109	6.5
Male sterilization	1	0.1
**Traditional method**	22	1.3
Rhythm method	3	0.1
Withdrawal	19	1.1

The contraceptive prevalence rate was 27.4% (n = 462) in the sampled population. Furthermore, the overall use of modern contraceptive methods was observed to be slightly lower at 26.1% (n = 441). Among different methods, the most common short-term methods reported by the respondents were condoms (n = 87; 5.1%) followed by pills (n = 83; 5.0%) and injectables (n = 63; 3.7%). Moreover, the lactation amenorrhea method (LAM) use was reported by 6 women. However, one woman reported the use of standard days methods (SDM). Out of all the long-acting reversible contraceptive methods respondent’s reported using implants (n = 41; 2.4%) and IUDs (n = 12; 0.7%). Among irreversible/permanent methods of contraception, (n = 109; 6.5%) reported female sterilization and only one respondent reported male sterilization method use.

The prevalence of traditional methods was less than two percent amongst all surveyed women (n = 22; 1.3%). Among such practices, 1.1% (n = 19) of observations were recorded for the withdrawal method, which was found to be the most common, followed by the rhythm method (n = 3; 0.1%). Additionally, distribution of modern contraceptive methods among socio demographic characteristics attached as supporting information file (S2 Table).

### Predictors for use of modern contraceptive methods (MCM)

Table 3 shows the univariate and multivariate analysis of the predictors associated with the use of modern contraceptives.

**Table 3:** Predictors for use of modern contraceptive method (MCM) among Married Women of Reproductive age (MWRA- 15-49years).

Variable	Modern Contraceptive Use		Univariate Analysis		Multivariate Analysis	
	Yes	No	Unadjusted Odd Ratio	95% CI	Adjusted Odds Ratio	95% CI
**Number of living children**						
1	30 (6.8%)	230 (18.7%)	1	1	1	1
2	62 (14.3%)	210 (16.4%)	2.39*	1.42–4.02	2.64*	1.44–4.82
3	60 (12.4%)	203 (15.9%)	2.14*	1.28–3.56	2.64*	1.40–4.99
4 or more children	289 (66.6%)	600 (49.0%)	3.73*	2.27–6.14	5.23**	2.78–9.84
**Woman’s age (in years)**						
15–19	7 (2.2%)	44 (3.9%)	1	1		
20–24	35 (8.1%)	143 (12.0%)	1.18	0.56–2.50	-	-
25–29	58 (12.7%)	257 (19.1%)	1.17	0.55–2.48	-	-
30–34	117 (24.9%)	310 (25.0%)	1.74	0.80–3.78	-	-
35–39	119 (28.5%)	245 (19.6%)	2.55*	1.34–4.85	-	-
40 or above	105 (23.7%)	244 (20.5%)	2.02*	0.98–4.17	-	-
**Woman’s education**						
No education	289 (67.3%)	942 (76.0%)	1	1	1	1
Primary	79 (17.4%)	145 (12.3%)	1.59*	1.21–2.11	1.73**	1.26–2.36
Middle	20 (4.4%)	38 (3.0%)	1.62*	0.97–2.73	1.67	0.95–2.94
Secondary	20 (4.2%)	48 (3.8%)	1.24	0.71–2.15	1.53	0.82–2.86
Intermediate or above	33 (6.7%)	70 (4.8%)	1.57	0.93–2.67	1.81	1.01–3.25
**Husband’s education**						
No education	158 (38.0%)	501 (39.9%)	1	1	1	1
Primary	72 (15.6%)	253 (21.1%)	0.77	0.52–1.14	0.71	0.48–1.05
Middle	42 (8.9%)	67 (5.0%)	1.87*	1.20–2.93	1.69*	1.03–2.76
Secondary	61 (12.8%)	152 (12.2%)	1.09	0.73–1.62	0.95	0.63–1.43
Intermediate or above	107 (24.6%)	250 (20.0%)	1.27	0.91–1.77	0.96	0.65–1.41
**Wealth Index**						
Poorest	69 (14.4%)	266 (19.5%)	1	1	1	1
Poor	70 (16.8%)	266 (21.4%)	1.06	0.67–1.68	-	-
Middle	93 (21.8%)	246 (21.1%)	1.40	0.89–2.18	-	-
Rich	104 (24.7%)	234 (19.4%)	1.72*	1.05–2.82	-	-
Richest	105 (22.3%)	231 (18.7%)	1.62	0.97–2.68	-	-
**Sought ANC**	408 (92.6%)	1077 (87.4%)	1.79*	1.07–2.98	1.73*	1.07–2.79
**4 or more ANC Visits**	213 (48.8%)	521 (43.1%)	1.25*	0.95–1.77	-	-
**FP counseling during ANC Visit**	27 (5.9%)	50 (3.8%)	1.60*	0.74–3.44	-	-
**LHW Visited during pregnancy**	304 (69.0%)	865 (69.2%)	0.98	0.74–1.37	-	
**Skilled Birth Attendant**	360 (81.1%)	952 (76.7%)	1.30*	0.97–1.75	-	-
**Type of Delivery**						
Normal/Assisted Vaginal delivery	347 (80.0%)	1046 984.9%)	1	1		
C-Section	94 (20.0%)	197 (15.1%)	1.40*	1.04–1.88	-	-
**PNC visit of mother**	287 (63.0%)	707 (56.2%)	1.33*	1.00–1.75	1.46**	1.09–2.05
**LHW Visited after delivery**	300 (65.7%)	741 (57.9%)	1.39*	1.07–1.81	-	-
**LHW counseling for family planning as a part of PNC visit**	150 (33.6%)	253 (20.0%)	2.02*	1.56–2.62	1.83**	1.38–2.42

p value less than 0.2 was considered as statistically significant for univariate analysis to be included in the final model

*p value less than 0.05 and

**p value less than 0.001 was considered as statistically significant for multivariate analysis

#### Univariate analysis

The odds of using contraceptives among women with more than four children were four times higher than those with only one child (OR: 3.73; 95%CI: 2.27–6.14). The likelihood of use of contraceptives was two times higher for women aged 35–39 years compared to women aged below 19 years (OR: 2.55; 95%CI: 1.34–4.85). Women who had completed middle-level education were at least twice as likely to use modern contraceptives to delay pregnancy than those without education (OR: 1.62; 95%CI: 0.97–2.73). Similarly, husbands who had completed their middle-level education were at least two times more likely to use contraceptives compared to those who were uneducated (OR: 1.87; 95%CI: 1.20–2.93). The odds of modern contraceptive use among women who belonged to the rich wealth index were two times higher than women in the poorest wealth index category (OR: 1.722; 95%CI: 1.051–2.00).

Women who were using health facilities for maternal health services showed a significant increase in the odds of using contraceptives in their postpartum period compared to women who were not using health facilities. Mothers who sought antenatal care services had higher odds of adopting modern contraceptive methods (OR: 1.25; 95%CI: 0.956–1.77) as compared to those who did not have ANC visits. Similarly, mothers who had visited health facilities four or more times for ANC visits were 1.25 times more likely to use contraceptives in their postpartum period than those who had visited less than four times during their pregnancy.

Moreover, mothers who delivered by skilled birth attendant showed higher odds of using contraceptives than those who delivered at home (OR: 1.30; 95%CI: 0.99–1.79). The odds of use of contraceptives amongst women with cesarean birth were 1.4 times the odds of use of contraceptives amongst women who had either normal or assisted vaginal delivery (0R: 1.40; 95%CI: 1.04–1.88). Women who had a postnatal care also showed higher odds of using postpartum contraceptives than those who did not (OR: 1.33; 95%CI: 1.00–1.75). Lady Health Worker visits after delivery and for PNC were associated with a significant increase in the odds of the use of contraception (OR: 2.028; 95%CI: 1.56–2.62). Similarly, family planning counseling during antenatal care visits also showed a significant increase in the odds of the use of postpartum contraceptives compared to those who had not received any counseling (OR: 1.60; 95%CI: 0.74–3.44).

#### Multivariate analysis

The multivariate logistic model shows that women with four or more children are four times more likely to use contraceptives than those with only one child (AOR: 5.23; 95%CI: 2.78–9.84). Compared to the bivariate model, after controlling for other covariates, the odds of use of contraceptives were significantly higher among women who had completed primary education compared to uneducated women (AOR: 1.73; 95%CI: 1.26–2.36). The odds ratio remained significant for husbands who had attended middle level education in the multivariate model (AOR: 1.69; 95%CI: 1.03–2.76). The odds of a postnatal care of the mother were 1.4 times higher amongst mothers who had used contraceptives (AOR: 1.46; 95%CI: 1.09–2.05) as well as women who were visited by the Lady Health Worker in their postnatal period and were counseled on family planning (AOR: 1.83; 95%CI: 1.38–2.42).

## Discussion

This study assessed predictors for opting for voluntary modern contraceptive methods amongst women of reproductive age in Sindh. This was achieved through univariate and multivariate analysis for specific variables, including knowledge, practices, access, and use of reproductive and maternal health services. The results showed the number of children, mother’s education, husband’s education, postnatal care visits of mother, and LHW counseling for family planning as part of the PNC visit; were the most accurate predictors of modern contraceptive methods. These results provide valuable insights into designing family planning programs that are well-suited to the local context with its unique and challenging socio-cultural and patriarchal dynamics.

The study noted that the average number of living children was at least four children per MWRA; women with more than four children were more likely to use modern contraceptives. This showed a significant inclination of the sample population towards limiting their pregnancy rather than spacing between desired pregnancies, particularly in women aged more than 35 years for which majority of them opted for sterilization, a surgical procedure. Younger respondents, on the other hand, tended to prefer short-acting methods like condoms, pills, and injections. The desire for uptake of contraception by both younger and mature women as revealed in this study, is coherent with findings from studies conducted in Pakistan. A cross sectional study conducted on women’s decision-making autonomy as a facilitating factor for contraceptive uptake highlights women’s increasing acceptance and demand for contraceptive methods to avert unwanted pregnancies [13].

Despite this awareness and desire for contraceptive methods, the utilization of long-acting reversible contraceptives (LARC), such as intrauterine devices (IUDs) and implants, was not widely observed in this study. This finding is supported by a mixed methods study on reasons for low uptake on widely available modern contraceptive methods conducted in Pakistan. The study reported that married women of reproductive age perceive that long-acting methods are less effective and have severe and fatalistic side-effects, that could potentially reduce their fertility [25].

Conversely, contraception needs related to desire for spacing are predicted to be greater among younger women; however, the results show the reverse whereby contraceptive use amongst younger women was lower. This dilemma of high need but low use discerned in our results can be explained by findings from the literature. Studies on barriers to uptake in family planning and socio-demographic determinants of unmet need in Pakistan emphasize that younger women and couples are less likely to have a discussion about use of contraception due to a lack of knowledge, couple communication gaps in the initial years of marriage, and societal pressure to have children which leaves them, especially younger girls, less motivated to consider timely uptake of contraception [9, 12, 26]. Additionally, the prevalence of contraceptive use amongst respondents was almost similar to that reported in the national and regional statistical records [8]. The current study has further highlighted gaps between the awareness and practice of modern contraceptive methods. Though majority of the participants were aware of contraceptive methods, this did not translate into practice. Other studies conducted in Pakistan attribute this gap mainly to socio-cultural and societal barriers [25, 27].

Another significant element contributing to narrowing the gap between knowledge and application is the accessibility of contraceptive methods along with their consistent availability. The uninterrupted provision of these methods plays a pivotal role in addressing the issue of unmet needs and fostering the adoption of MCMs [28]. The importance of availability is emphasized as a major predictor of contraception usage in a quantitative study undertaken in Karachi, Pakistan. The study iterates that a lack of contraceptive commodities leads to high rates of discontinuation and reduced effectiveness of contraception and more broadly family planning efforts [10].

Moreover, other negative attitudes and misconceptions, including fear of side effects and a preference for a male baby, continue to contribute to low uptake of contraceptives. This is echoed in various qualitative and mixed methods studies conducted on family planning in Pakistan that reiterate the desire for a son, specifically in rural areas, as a key hinderance to uptake of contraception. These studies also link the lack of community knowledge and awareness of contraception with myths and ill-founded suspicions regarding side effects of contraception, including death, that continue to be spread through social communication networks within local neighborhoods and communities [9, 29–32].

Interestingly, the study found that amongst subgroups in the sample population, the contraceptive prevalence rate was higher amongst women falling within the rich quintile. This finding is similar to other studies, both in the local and global literature, that explore and validate a strong positive association between increasing level of wealth quintile and increased ease of access to FP information and contraceptive services as well as sustained use of MCMs [33, 34].

Interestingly, women educated at the primary level were more likely to opt for modern contraceptive methods than women who had attained comparatively higher education as opposed to previous studies [31, 34]. Furthermore, in univariate analysis, contraceptive use showed an increase with increasing levels of males’ education. These findings are compounded by the literature that describes a comprehensive range of studies on family planning that emphasize the role of male engagement in improving uptake, specifically through (i) enabling male partners / spousal support for adopting a contraceptive method and (ii) involving their female partners in decisions regarding family size, reproductive health, and use of an MCM [26, 35–38].

This study’s most important finding was identifying postnatal care (PNC) as the most significant predictor for opting for family planning. Respondents who sought PNC were more likely to use modern contraceptive methods. Evidence in the literature has endorsed that postnatal counseling before discharge significantly improves motivation for and practice of contraceptive uptake [25, 28–30]. This study also observed that Lady Health Worker’s counselling delivered during a PNC visit increased the likelihood of family planning utilization. The probable cause of this finding can be linked to the preference of the local community for Lady Health Workers to discuss family planning matters on a one-to-one basis at the household level while maintaining confidentiality [39]. Moreover, Lady Health Workers also provide family planning commodities like condoms and pills during their routine visits. Consistent with our results, referral to a health care facility through LHW also increases postpartum utilization of family planning. Therefore, the Lady Health Worker Program strongly influences women’s fertility choices and their use of sexual and reproductive health care services [30, 40].

Our study population reported a lower prevalence of family planning counseling received during ANC visit. At the same time, these visits still influenced positive behaviors towards modern contraceptive methods. In addition to this, family planning exposure was seen to be associated with subsequent antenatal care visits, a higher number of deliveries performed by skilled professionals, and a greater chance of delivery at a health facility [41]. Earlier studies have shown a positive relationship between the delivery of high-quality antenatal family planning counseling services and increased contraceptive use [4, 42].

Another study conducted in a similar setting as our study sites, concluded that in communities where a higher proportion of women received quality antenatal care with an explanation of the importance of postpartum family planning and where discussion of birth spacing was more common, there was significantly higher contraceptive use particularly for LARCs [41, 43]. In light of these findings, this study recommends integrating family planning with maternal, newborn, and child health services and using community engagement as a key mechanism of service delivery. In that, these integrated services can be delivered through ongoing antenatal and postnatal care visits conducted by well-trained Lady Health Workers that incorporate a renewed focus on providing family planning counselling, information, referral and services for women, young couples as well as men [44–47].

These studies were short-term funded programs highlighting the need for sustainable programs and scaling up existing resources. Thus, multiple determinants then affect contraceptive uptake in the context of Sindh, and strategically addressing these are imperative in improving MCM prevalence rates.

Addressing these determinants requires an inter-sectoral approach that integrates various local, regional and national sectors such as policymaking, programming, education, health and development. By building evidence for predictors that affect contraceptive uptake, this study provides an important basis for informing, rethinking, and strengthening the current public health agenda and policies that focus on improving access, availability, quality and affordability of contraceptive services, specifically to a greater proportion of high-risk populations within the country.

## Conclusion

According to the aim of this study to assess predictors associated with the uptake of MCMs in rural Sindh, this study showed that the voluntary uptake of modern contraceptive methods is higher in women receiving antenatal care, postnatal care at facility and skilled-based delivery, family planning counseling Lady Health Workers visits after delivery. Education was also seen as an important predictor as the odds of use of contraceptives were significantly higher amongst women with primary education as compared to women with no education.

The findings also revealed that existing family size is an important predictor of uptake, in that, women with four or more children are more likely to use contraceptives than those with one child. The main purpose of using MCMs is for limiting pregnancies rather than maintaining birth-spacing and utilization increases with age. This is an important finding that can be used to inform behavioral change interventions to improve outcomes.

## Supporting information

S1 Table Selection of districts using Propensity Score Matching (PSM) from 23 rural districts of Sindh. (PDF)

S2 Table Distribution of modern contraceptive methods among sociodemographic characteristics. (PDF)
