# Supplementary material for: Predictors of voluntary uptake of modern contraceptive methods in rural Sindh, Pakistan
Source: PLOS Glob Public Health. 2024 Apr 4;4(4):e0002419. doi: 10.1371/journal.pgph.0002419 (PMC10994370; doi:10.1371/journal.pgph.0002419)
Supplement: S1 Table — (PDF) [file pgph.0002419.s001.pdf]

S1_Table. Selection of Districts using Propensity Score Matching (PSM) from 23 rural districts of Sindh

SN	Districts	UMN	CPR	mCPR	HDI	FIMM	DSBA	ANC	Propensity Score(PSM)
1	**Badin**	17.9	28.1	28.1	41	45.6	61.9	82	**0.36**
2	Sanghar	21.7	24.9	24.9	49	59.4	59.4	76	0.19
3	**Matiari**	25.8	32.4	30.6	57	68	65.5	85.7	**0.31**
4	Qamber Shahdadqot	19.3	18.4	18.2	46	22.6	52.4	70.3	0.06
5	Kashmore	26.1	16	15.7	47	9.7	35.9	63.5	0.12
6	Jacobabad	22.8	19.9	16.9	44	18	51.9	59.5	0.00
7	Larkana	20.6	26.7	24.3	62	41	55.8	70.3	0.04
8	Shikarpur	26.9	11.3	10.8	52	32	47.3	72.6	0.02
9	Ghotki	24.5	22.2	21.4	51	19.6	43.1	55.4	0.09
10	Sukkur	20.9	27.2	26.9	66	27.9	72.8	69.4	0.14
11	Khairpur	22.4	19.7	18.7	56	29.8	55.5	90.8	0.17
12	Naushahro Feroz	21.7	20.7	18.5	67	62.5	53.4	75.8	0.01
13	Shaheed Benazirabad	22.8	23.9	22	57	36.4	63.7	76.8	0.04
14	Dadu	21.8	19.7	18.8	63	18.9	50.2	74.5	0.10
15	Jamshoro	20.7	21.9	21.4	57	68.5	57.2	75.3	0.06
16	Hyderabad	17.5	33.3	28.9	72	61.3	90.6	95	0.01
17	Tando Allahyar	21.4	28.8	26.1	53	63.8	67.4	91.7	0.09
18	Tando Muhammad Khan	21.2	28.5	26.4	38	32.1	71.8	79.9	0.08
19	Sujawal	26.2	15.9	15.3	33	40.2	52.6	79.3	0.06
20	Thatta	22.6	19.7	15.9	38	37.4	59.4	74.1	0.00
21	Mirpurkhas	23.9	24.5	21.9	43	38.1	51.8	61.9	0.02
22	Umerkot	22.4	19.2	19	32	60.1	37.3	55.8	0.04
23	Tharparkar	30.9	12.1	11.7	23	31.3	20.7	30.6	0.01
