# Supplementary material for: Predictors of voluntary uptake of modern contraceptive methods in rural Sindh, Pakistan
Source: PLOS Glob Public Health. 2024 Apr 4;4(4):e0002419. doi: 10.1371/journal.pgph.0002419 (PMC10994370; doi:10.1371/journal.pgph.0002419)
Supplement: S2 Table — (PDF) [file pgph.0002419.s002.pdf]

S2_Table. Distribution of MCM among sociodemographic characteristics

	Female Sterilization	Male Sterilization	IUD (Intrauterine Device)	Injection	Implants	Pills	Condom	SDM (Standard Days Method)	Lactation amen method (LAM)	Rhythm method	Withdrawal
**No of children**											
1	0 (0%)	0 (0%)	0 (0%)	7 (2.6%)	0 (0%)	5 (1.6%)	16 (6.4%)	0 (0%)	0 (0%)	1 (0.3%)	1 (0.3%)
2	4 (1.7%)	0 (0%)	1 (0.3%)	21 (8.3%)	3 (0.9%)	16 (6.1%)	15 (5.7%)	0 (0%)	0 (0%)	1 (0.3%)	2 (0.7%)
3	6 (2.3%)	0 (0%)	1 (0.4%)	20 (7.6%)	4 (1.5%)	12 (4.2%)	12 (4.1%)	1 (0.3%)	0 (0%)	0 (0%)	1 (0.3%)
4 or more children	99 (11.5%)	1 (0.1%)	10 (1.1%)	65 (7.5%)	29 (3.3%)	29 (3.3%)	44 (4.4%)	0 (0%)	1 (0.1%)	1 (0.3%)	13 (0.9%)
**Respondent's age**											
15-19	0 (0%)	0 (0%)	0 (0%)	3 (6.9%)	1 (1.5%)	2 (6.7%)	1 (1.6%)	0 (0%)	0 (0%)	0 (0%)	0 (0%)
20-24	0 (0%)	0 (0%)	0 (0%)	13 (7.5%)	1 (0.6%)	5 (2.2%)	15 (8.5%)	0 (0%)	0 (0%)	0 (0%)	0 (0%)
25-29	7 (2.3%)	0 (0%)	2 (0.6%)	14 (4.5%)	1 (0.4%)	15 (4.8%)	13 (4.8%)	1 (0.3%)	0 (0%)	0 (0%)	4 (1.2%)
30-34	20 (5%)	0 (0%)	2 (0.4%)	37 (8.7%)	11 (2.3%)	16 (3.2%)	25 (5.2%)	0 (0%)	0 (0%)	3 (0.7%)	7 (1.6%)
35-39	48 (13.8%)	1 (0.3%)	3 (1.2%)	22 (6%)	7 (2.1%)	13 (4.4%)	22 (5.3%)	0 (0%)	0 (0%)	0 (0%)	5 (1.3 %)
40 or above	34 (9.8%)	0 (0%)	5 (1.1%)	24 (7.4%)	15 (4.3%)	11 (2.5%)	11 (2.6%)	0 (0%)	1 (0.2%)	0 (0%)	4 (1.1%)
**Respondent'sEducation**											
No education	70 (6.3%)	0 (0%)	10 (0.8%)	84 (7%)	30 (2.5%)	40 (3.2%)	38 (2.8%)	1 (0.1%)	1 (0.1%)	3 (0.2%)	10(0.8%)
Primary	26 (10.1%)	1 (0.5%)	2 (0.8%)	18 (7.9%)	3 (1.3%)	12 (5.4%)	16 (6.9%)	0 (0%)	0 (0%)	0 (0%)	6 (2.6%)
Middle	6 (9.2%)	0 (0%)	0 (0%)	7 (13.1%)	0 (0%)	2 (2.8%)	4 (6.7%)	0 (0%)	0 (0%)	0 (0%)	0 (0%)
Secondary	3 (5%)	0 (0%)	0 (0%)	1 (1.2%)	1 (1.2%)	2 (2.7%)	12 (16%)	0 (0%)	0 (0%)	0 (0%)	3(4.4%)
Intermediate or above	4 (3.9%)	0 (0%)	0 (0%)	3 (3%)	2 (1.9%)	6 (5.4%)	17 (18.2%)	0 (0%)	0 (0%)	0 (0%)	0 (0%)
**Huband's Education**											
No education	34 (6.1%)	1 (0.2%)	7 (0.9%)	54 (8.8%)	19 (2.8%)	25 (3.9%)	14 (1.9%)	0 (0%)	0 (0%)	0 (0%)	2 (0.3%)
Primary	16 (5%)	0 (0%)	1 (0.3%)	21 (5.9%)	6 (1.7%)	7 (2%)	16 (4.3%)	0 (0%)	0 (0%)	1 (0.3%)	2 (0.5%)
Middle	9 (8.8%)	0 (0%)	1 (1%)	10 (9%)	3 (3.8%)	6 (5.2%)	10 (8.4%)	0 (0%)	0 (0%)	0 (0%)	1 (0.8%)
Secondary	17 (7.4%)	0 (0%)	2 (0.7%)	11 (5%)	7 (3.3%)	7 (3.3%)	13 (5.6%)	1 (0.4%)	1 (0.4%)	0 (0%)	4 (1.8%)
Intermediate or above	33 (8.9%)	0 (0%)	1 (0.8%)	17 (5.2%)	1 (0.3%)	17 (4.3%)	34 (9.5%)	0 (0%)	0 (0%)	0 (0%)	3 (0.8%)
**Wealth Index**											

Poorest	15 (4.3%)	1 (0.4%)	2 (0.5%)	28 (8.4%)	6 (2%)	11 (3.7%)	5 (1.3%)	0 (0%)	0 (0%)	0 (0%)	2 (0.7%)
Poor	15 (4.5%)	0 (0%)	3 (0.7%)	22 (7.9%)	9 (2.7%)	8 (2.6%)	6 (1.5%)	1 (0.3%)	0 (0%)	0 (0%)	2 (0.7%)
Middle	22 (7.3%)	0 (0%)	3 (0.8%)	30 (8.5%)	10 (2.7%)	14 (3.7%)	9 (2.4%)	0 (0%)	1 (0.2%)	0 (0%)	5 (1.4%)
Rich	30 (9.6%)	0 (0%)	2 (1%)	21 (5.7%)	7 (2.2%)	15 (4.1%)	25 (7.5%)	0 (0%)	0 (0%)	1 (0.3%)	3 (0.9%)
Richest	27 (7.7%)	0 (0%)	2 (0.6%)	12 (4%)	4 (1.1%)	14 (3.8%)	42 (11.4%)	0 (0%)	0 (0%)	2 (0.6%)	7 (2.0%)
